# Altered frontal white matter microstructure is associated with working memory impairments in adolescents with congenital heart disease: A diffusion tensor imaging study

**DOI:** 10.1016/j.nicl.2019.102123

**Published:** 2019-12-16

**Authors:** Melanie Ehrler, Beatrice Latal, Oliver Kretschmar, Michael von Rhein, Ruth O'Gorman Tuura

**Affiliations:** a Child Development Center, University Children's Hospital Zurich, Switzerland; b Children's Research Center, University Children's Hospital Zurich, Switzerland; c Department of Pediatric Cardiology, University Children's Hospital Zurich, Switzerland; d Developmental Pediatrics, Center for Social Pediatrics, Cantonal Hospital Winterthur, Switzerland; e Center for MR Research, University Children's Hospital Zurich, Switzerland

**Keywords:** Diffusion tensor imaging, Congenital heart disease, Working memory, Cognition, Neurodevelopment, ADHD, attention deficit/hyperactivity disorder, CHD, congenital heart disease, FA, fractional anisotropy, TR/TE, repetition time/echo time, SES, socioeconomic status, TFCE, threshold-free cluster enhancement, TBSS, tract based spatial statistics, WISC-IV, Wechsler intelligence scale for Children 4th edition

## Abstract

Children and adolescents with congenital heart disease (CHD) are at risk for mild to moderate cognitive impairments. In particular, impaired working memory performance has been found in CHD patients of all ages. Working memory is an important domain of higher order cognitive function and is crucial for everyday activities, with emerging importance in adolescence. However, the underlying neural correlate of working memory impairments in CHD is not yet fully understood.

Diffusion tensor imaging and tract based spatial statistics analyses were conducted in 47 adolescent survivors of childhood cardiopulmonary bypass surgery (24 females) and in 44 healthy controls (24 females) between 11 and 16 years of age (mean age = 13.9, SD = 1.6). Fractional anisotropy (FA) of white matter diffusion was compared between groups and was correlated with working memory performance, derived from the Wechsler Intelligence Scale for Children-IV.

CHD patients had significantly poorer working memory compared to controls *(p* = 0.001). Widespread bilateral reduction in FA was observed in CHD patients compared to healthy controls (threshold-free cluster enhancement (TFCE) corrected *p* < 0.05). This reduction in FA was present both in cyanotic and acyanotic CHD patients compared to healthy controls (both *p* < 0.001). The FA reduction in the frontal lobe, mainly in the forceps minor, was associated with poorer working memory performance in both patients with CHD and healthy controls (TFCE corrected *p* < 0.05).

The current findings underline that in CHD patients, irrespective of disease severity, disrupted or delayed maturation of white matter may persist into adolescence and is associated with working memory impairments, particularly if present in the frontal lobe. Adolescence, which is a crucial period for prefrontal brain maturation, may offer a window of opportunity for intervention in order to support the maturation of frontal brain regions and therefore improve higher order cognitive function in patients with CHD.

## Highlights

• Patients with congenital heart disease (CHD) are at risk for lower working memory.
• CHD patients showed widespread alterations in white matter microstructure using DTI.
• Alterations in frontal white matter were associated with working memory problems.
• This association was independent of CHD type, IQ and group.

## 1 Introduction

It is well recognized that patients with severe congenital heart disease (CHD) undergoing open-heart surgery are at risk for mild to moderate cognitive impairments (Latal, 2016). Previous studies have shown that working memory impairments occur more frequently in CHD patients than in the typically developing population during childhood (Calderon et al., 2010) and adolescence (Bellinger et al., 2015b; Schaefer et al., 2013), and may persist into adulthood (Ilardi et al., 2017; Murphy et al., 2015). These impairments manifest themselves in both the school and home settings and are therefore of particular relevance (Cassidy et al., 2014; Sanz et al., 2017).

Working memory is the ability to temporally maintain and manipulate a limited amount of information (Baddeley, 2012). Working memory abilities are predictive for general intelligence (Peterman and Peterman, 2008), academic attainment (Alloway and Alloway, 2010) and are crucial for the successful performance of many daily life activities (Osaka et al., 2012).

In healthy children and adolescents, the maturation of fronto-parietal (Krogsrud et al., 2018; Østby et al., 2011; Peters et al., 2012; Vestergaard et al., 2010), fronto-temporal (Krogsrud et al., 2018), and frontal interhemispheric (Krogsrud et al., 2018) white matter structures is associated with improving working memory performance. In preterm-born adolescents, a population with a similar profile of cognitive deficits as in CHD patients (Easson et al., 2018), reduced anisotropy of white matter microstructure was observed compared to term-born children and was associated with working memory impairments (Vollmer et al., 2017).

The etiology underlying working memory impairments in CHD patients has not been well described. Few studies have linked structural and functional changes in brain development to reduced working memory performance. In particular, an fMRI study of King and colleagues showed functional differences between adolescents with CHD and healthy controls during a working memory task. These findings were mainly located in the frontal-parietal brain network (King et al., 2016). Evidence for structural alterations has emerged from studies reporting lower total brain and hippocampal volumes related to lower working memory performance (Latal et al., 2016; Von Rhein et al., 2014). Further, alterations of white matter microstructures were associated with impaired general cognitive function, such as IQ (Watson et al., 2018), executive function (Rollins et al., 2014), attention, and learning (Brewster et al., 2015) in adolescents with CHD. However, to date no study has investigated the relationship between working memory, as a distinct cognitive domain, and white matter microstructure in CHD patients.

We thus hypothesized that white matter microstructure within fronto-parietal, fronto-temporal and frontal interhemispheric networks would be altered in adolescents with CHD, and that these alterations would be related to working memory impairments.

## 2 Materials and methods

### 2.1 Sample

The studied patients originate from a sample of patients with different types of CHD who underwent full-flow cardiopulmonary bypass surgery at the University Hospital Zurich between 1995 and 1998 (age at surgery in years: median = 0.9, range = 0 - 5.6). Inclusion criteria were: parents fluent in German, age between 6 and 16 years at the time of the first neurodevelopmental examination and no genetic syndrome or any other congenital or neurological disease (for further details see Von Rhein, Dimitropoulos, Valsangiacomo Buechel, Landolt, and Latal, 2012). Of 117 subjects who took part in a neurodevelopmental examination at school-age, we excluded those who were 17 years of age or older at the time of the current study and those who did not meet the inclusion criteria for a cerebral MRI examination. Of 78 eligible participants, 23 refused to take part in the study and two could not been contacted. Therefore, 53 patients were assessed in the current study.

In three of the 53 participants the DTI sequence was not conducted and three participants had to be excluded due to extensive movement artifacts in the MRI. Thus, a total of 47 patients with CHD and 44 healthy controls with comparable socio-economic status and sex were included in the study. Healthy controls were enrolled for this study (*n* = 10) or had participated in another study (*n* = 34) using the same DTI and cognitive test protocols. Control participants were free from any chronic or neurological disease or brain lesions.

The study was approved by the ethics committee of the University Children's Hospital Zurich and all the participants, as well as their parents or primary care givers gave written informed consent prior to the study participation.

### 2.2 Outcome assessment

IQ was assessed using the German version of the Wechsler intelligence scale for Children 4th edition (WISC-IV; Peterman and Peterman, 2008). The main outcome of this analysis, working memory performance, was evaluated using the two subtests *digit span* and *letter-number sequencing*. According to the manual of the WISC-IV, a composite score for working memory was built adjusting for sex and age. During the *digit span* test, participants were asked to repeat a series of numbers in the same and in reverse order to that read aloud by the examiner. The number of digits was increased step-wise up to nine digits as long as the participant was able to repeat at least one of two sequences correctly. During the *letter-number sequencing* test*,* participants were read increasing series of letters and numbers and were then asked to repeat them in the correct numerical and alphabetic order (1 to 10, A to Z). Both tests were designed in order to measure the ability of holding and manipulating verbal information. Socio-economic status (SES) was estimated by means of the sum of a six-point scale of maternal education and paternal occupation resulting in an SES range from two to 12 (Largo et al., 1989). Participants were examined by an experienced pediatrician, who was aware of their medical condition but not of their brain MRI findings.

### 2.3 DTI acquisition and processing

Brain MRI was performed with a 3.0 Tesla whole-body system (SignaTwinspeed HD.xt, GE Healthcare, Milwaukee, WI). A diffusion tensor imaging sequence was acquired, oriented parallel to the anterior commissure—posterior commissure plane, with parameters: repetition time/echo time (TR/TE) = 1250/93 msec; flip angle = 90°; acquisition matrix = 128 × 128 (reconstructed to 256 × 256); field of view = 220 mm, slice thickness 3 mm; voxel size = 0.86 × 0.86 × 3mm^3^. A total of 21 diffusion-weighted gradient directions were acquired with *b* = 1000s/mm2 and five interleaved non-diffusion weighted images with *b* = 0 s/mm2.

DTI data was processed using FSL Software on Linux (Smith et al., 2004). First, correction was done for eddy current artifacts (Andersson and Sotiropoulos, 2016) followed by brain extraction using BET (Brain Extraction Tool, Smith, 2002). Subsequently the diffusion tensors were fitted at each intracerebral voxel in order to calculate maps of the fractional anisotropy (FA) for each participant.

FA images were aligned into (1 × 1 × 1 mm3) MNI152 standard space using tract-based spatial statistics (TBSS; Smith et al., 2006), and the group mean FA map was generated. The white matter FA skeleton was then generated from the mean FA map and thresholded using an FA cutoff of 0.2. FA values range from zero to one, in which greater values correspond to higher microstructural white matter integrity.

### 2.4 Statistical analysis

In a first step, the sample characteristics, IQ, working memory performance, age and SES, were examined with a two-tailed *t*-test and sex was examined with a chi-squared test in order to investigate group differences. Median and range of various cardiac parameters were calculated in patients. School performance of patients was investigated with regard to receiving school support or completing an additional year of school as a part of regular schooling.

Subsequently, FA values were compared on a voxel-wise basis between the two groups, CHD patients and healthy controls, and were associated with working memory performance, adjusting for group. Post hoc, working memory was associated with FA for both groups separately. Statistical inference testing was performed using the (non-parametric) permutation testing methods implemented in the FSL Randomize tool to account for the non-normal Gaussian distribution of FA values. The significance threshold was set to α = 0.05 and was corrected for multiple comparisons using threshold-free cluster enhancement (TFCE). Significant white matter structures were identified using the JHU ICBM-DTI-81 White Matter, the JHU White Matter Tractography and the Harvard-Oxford Subcortical Structural atlases.

Post hoc analyses were conducted for significant findings, after generating a mask for the significant clusters using fslmaths, and extracting the mean FA value of the resulting significant clusters for each participant. Sex and age were included as covariates in all models. As a first post hoc analysis, mean FA values were compared between healthy controls and patients having an acyanotic and a cyanotic CHD, respectively, in order to investigate whether the presence of a cyanotic heart defect could better explain reduction in white matter microstructure (Model 1: dependent variable = FA; independent variable = subgroups (cyanotic, acyanotic, control); covariates = sex, age). In addition, other subgroups of cardiac factors were tested, namely neonatal CPB surgery, number of CPB surgeries, or side of CHD. As a second post hoc analysis, a regression model with mean FA as dependent variable was tested including not only working memory performance but also IQ as independent variables, to ensure that the association between working memory performance and FA is independent of IQ (Model 2: dependent variable = FA; independent variables = working memory, IQ; covariates = sex, age). Furthermore, an interaction between type of CHD (cyanotic, acyanotic) and working memory was tested in a separate linear regression model (Model 3: dependent variable = FA; independent variables = working memory, subgroups (cyanotic, acyanotic, control), interaction (working memory x subgroups); covariates = sex, age). Five participants of the control group were excluded from the analyses including working memory performance because at least one working memory sub-test was missing in these individuals. All post-hoc analyses of mean FA in the full sample, as well as baseline comparison were performed using the computing environment R version 3.5.3 (R Core Team, 2018) with an α = 0.05. The STROBE guidelines for reporting observational studies were used.

## 3 Results

### 3.1 Participant characteristics

Participant characteristics of patients with CHD and healthy controls are given in Table 1. Medical characteristics of patients are given in Table 2. Three patients with a double inlet left ventricle (DILV) underwent a Fontan palliation. The participating patients did not differ to the patients lost-to follow up with regard to demographic or surgical characteristics, but had fewer neurological abnormalities and better cognitive functioning (see Von Rhein et al., 2014). Patients and controls did not significantly differ in regard to sex, age and SES, although controls showed a trend towards higher SES.

**Table 1:** Participant characteristics and cognitive data stratified by group.

Variables	CHD Patients (*n* = 47)	Controls (*n* = 44)	*P*
Age^a^	13.7 (1.6)	13.9 (1.8)	0.56
Male sex^b^	23 (48.9)	20 (45.5)	0.93
SES^a^	7.7 (2.1)	8.6 (1.5)	>0.05
IQ at assessment^a^	104.9 (16.6)	112.5 (10.3)	0.01
Working memory^a^	94.1 (13.1)	103.4 (12.2)	<0.01
Receiving learning support in school^b^	14 (29.8)		
Receiving reading support in school^b^	8 (17.0)		
Receiving math support in school^b^	9 (19.1)		
Completed an additional year of regular schooling^b^	16 (34.0)		

SES socioeconomic status (range 2–12).

a Mean (standard deviation).

b Number of individuals (%). *P* = uncorrected P-value. Data on schooling was not available for controls.

**Table 2:** Medical characteristics of patients.

Variables	CHD Patients (*n* = 47)
Acyanotic CHD^b^	23 (48.9)
Atrial or ventricular septal defect^b^	16 (34.0)
Aortic stenosis^b^	3 (6.4)
Pulmonary stenosis^b^	2 (4.3)
Aortic coarctation^b^	1 (2.1)
Shone complex^b^	1 (2.1)
Cyanotic CHD^b^	24 (51.1)
Transposition of the great arteries^b^	10 (21.2)
Tetralogy of Fallot^b^	5 (10.6)
Pulmonary atresia^b^	2 (4.3)
Double inlet left ventricle^b^	3 (6.4)
Total anomalous pulmonary venous connection^b^	2 (4.3)
Tricuspid atresia^b^	1 (2.1)
Truncus arteriosus^b^	1 (2.1)
Age at first surgery (y)^c^	0.9 (0–5.6)
Number of patients with neonatal surgery^b^	9 (19.1)
Weight at first surgery (kg)^c^	7.8 (2.8–19.5)
Duration of extracorporeal circulation (min)^c^	89 (5–149)
Duration of aortic cross-clamping (min)^c^	39 (13–83)
Circulatory arrest time (min)^c^	0 (0–18)
Length of intensive care unit stay (d)^c^	8 (1–53)
Length of hospital stay (d)^c^	16 (7–71)
Total number of CPB surgeries^c^	1 (1–3)

b Number of individuals (%).

c Median (range).

Both IQ (*t*(84) = 2.51, *P <* 0.014, *CI-95* = 1.58 to 13.75) and working memory performance (*t*(84) = 3.38, *P <* 0.001, *CI-95* = 3.84 to 14.81) were significantly lower in CHD patients compared to healthy controls. Working memory performance was not significantly poorer in those patients with cyanotic CHD (*t*(45) = −0.22, *P <* 0.83, *CI-95* = −8.66 to 6.95) or in those with more than one bypass surgery (*t*(45) = 1.83, *P <* 0.07, *CI-95* = −0.80 to 16.47). Also, those receiving learning support (*t*(45) = −0.72, *P* = 0.5, *CI-95* = −10.82 to 5.14) or those who have completed an additional year of regular schooling did not show worse performance (*t*(45) = −1.46, *P* = 0.2, *CI-95* = −13.86 to 2.23).

### 3.2 Comparison of white matter microstructure between patients with CHD and healthy controls

**Fig. 1:**
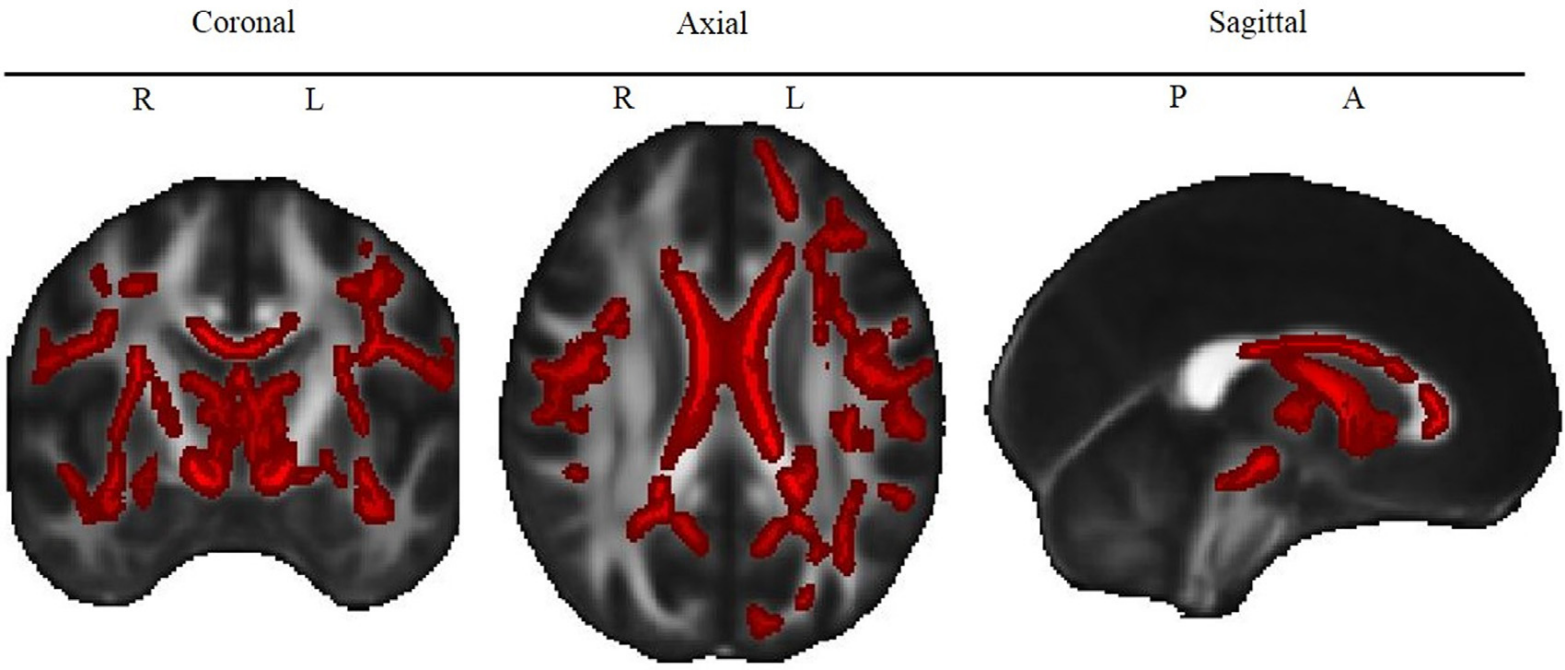
Lower FA in CHD patients compared to controls.

TBSS analyses revealed lower FA in CHD patients than healthy controls in the Corpus callosum, forceps minor, forceps major, fornix and thalamus and in the bilateral cerebral peduncle, cingulum, corona radiata, corticospinal tract, external and internal capsule, inferior and superior (only right) fronto-occipital fasciculus, inferior and superior longitudinal fasciculus, thalamic radiation and uncinate fasciculus (see Table 3 and Fig. 1).

**Table 3:** White matter structures with significantly lower FA in patients with CHD than in healthy controls.

White matter Structure	P*	X	Y	Z
Cerebral peduncle (L)^b^	0.01	−5	−18	−21
Cerebral peduncle (R)^b^	0.01	11	−20	−21
Cingulum (L)^a^	0.01	−10	29	14
Cingulum (R)^a^	0.02	15	−56	30
Corona radiata – anterior (L)^b^	0.02	−16	35	5
Corona radiata – anterior (R)^b^	0.02	18	23	−13
Corona radiata – posterior (L)^b^	0.04	−21	−48	28
Corona radiata – posterior (R)^b^	0.02	21	−31	39
Corona radiata – superior (L)^b^	0.02	−28	−7	19
Corona radiata – superior (R)^b^	0.01	19	−19	37
Corpus callosum – body^b^	0.01	4	18	17
Corpus callosum – genu^b^	0.01	−12	29	13
Corpus callosum – splenium^b^	0.02	27	−54	15
Corticospinal tract (L)^a^	0.01	−5	−18	−21
Corticospinal tract (R)^a^	0.01	11	−20	−21
External capsule (L)^b^	0.02	−34	−12	−12
External capsule (R)^b^	0.02	37	−11	−13
Forceps major^a^	0.02	28	−63	14
Forceps minor^a^	0.01	−12	29	13
Fornix (column and body of fornix)^b^	0.01	0	4	3
Fronto-occipital fasciculus – inferior (L)^a^	0.02	−32	38	1
Fronto-occipital fasciculus – inferior (R)^a^	0.02	40	−13	−15
Fronto-occipital fasciculus – superior (R)^b^	0.02	22	2	19
Inferior longitudinal fasciculus (L)^a^	0.02	−39	−9	−21
Inferior longitudinal fasciculus (R)^a^	0.02	40	−13	−15
Internal capsule – anterior (L)^b^	0.02	−10	−1	−1
Internal capsule – anterior (R)^b^	0.02	13	12	−4
Internal capsule – posterior (L)^b^	0.01	−11	−5	−4
Internal capsule – posterior (R)^b^	0.02	18	−5	7
Internal capsule – retrolenticular (L)^b^	0.02	−37	−26	−3
Internal capsule – retrolenticular (R)^b^	0.02	30	−23	2
Superior longitudinal fasciculus – temporal (L)^a^	0.02	−52	−5	16
Superior longitudinal fasciculus – temporal (R)^a^	0.01	35	−34	33
Superior longitudinal fasciculus (L)^a^	0.02	−46	6	5
Superior longitudinal fasciculus (R)^a^	0.01	35	−34	33
Thalamic radiation – anterior (L)^a^	0.01	−4	−11	2
Thalamic radiation – anterior (R)^a^	0.01	2	−15	3
Thalamic radiation – posterior (L)^b^	0.03	−34	−61	3
Thalamic radiation – posterior (R)^b^	0.02	28	−63	14
Thalamus (L)^c^	0.01	−4	−11	2
Thalamus (R)^c^	0.01	13	−16	10
Uncinate Fasciculus (L)^a^	0.02	−32	38	1
Uncinate Fasciculus (R)^a^	0.01	38	−10	−15

For each structure, we report.

⁎ TFCE corrected p-values and cluster center coordinates (X, Y, Z) of the most significant cluster. Only clusters with >30 voxels were considered. Structures were extracted from three atlases:

a JHU White Matter Tractography Atlas,

b JHU ICBM-DTI-81 White Matter Atlas,

c Harvard-Oxford Subcortical Structural Atlas. *L* = left hemisphere, *R* = right hemisphere.

White matter structures that show significantly lower FA in CHD patients compared to healthy controls. Red color indicates voxels for which FA is significantly (TFCE corrected *P* < 0.05) lower in the CHD group compared to controls. *L* = Left, *R* = Right, *P* = Posterior, *A* = Anterior. See Supplementary Figure 1 for the map of the different tracts.

In a linear regression model (Model 1, see Section 2.4), mean FA of all significant clusters was lower in both cyanotic (*β* = −0.030, *t*(86) = −5.442, *P <* 0.001, *CI-95* = −0.041 −0.019) and acyanotic (*β* = −0.019, *t*(86) = −3.574, *P <* 0.001, *CI-95* = −0.029 −0.008) CHD than in healthy controls. FA values did not differ significantly between cyanotic and acyanotic CHD (*β* = - 0.009, *t*(86) =, *P* *=* *=0.20, CI-95* = 0.004 to 0.013). FA was neither associated with sex (*β* = −0.000, *t*(86) = 0.017, *P* *=* *=*0.986, *CI-95* = −0.008 0.009) nor with age at examination (*β* = 0.001, *t*(83) = −0.827, *P* *=* *=*0.37, *CI-95* = −0.004 0.002) within the sample of CHD adolescents. The linear regression model explained 24% of the variance in FA (adjusted *R^2^* = 0.243). In addition, we also tested whether other cardiac variables, such as neonatal CPB surgery, number of CPB surgeries, or side of CHD were associated with FA. None of these variables was significant (see Supplementary Table 1). However, these analyses were underpowered due to the small size of the subsamples.

### 3.3 Association of working memory performance and white matter microstructure

**Fig. 2:**
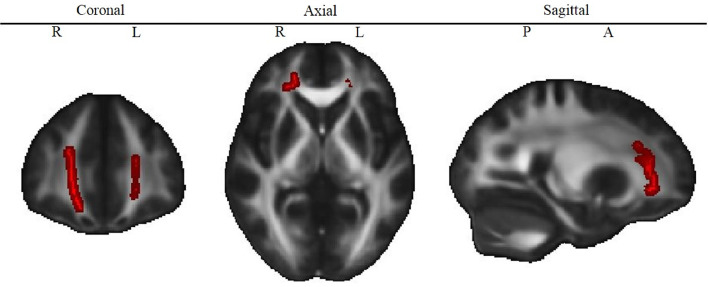
Correlation of FA and working memory.

Reduced working memory performance was associated with reduced FA in the bilateral forceps minor and the right frontal part of the uncincate fasciculus and the inferior fronto-occipital fasciculus (Table 4 and Fig. 2). This association was found in both groups (patients with CHD, healthy controls).

**Table 4:** White matter structures that significantly correlate with working memory performance in patients and controls.

White matter Structure	P*	X	Y	Z
Forceps minor	0.04	21	37	2
Inferior fronto-occipital fasciculus (R)	0.04	19	39	1
Uncinate fasciculus (R)	0.04	20	44	1

⁎ TFCE corrected p-values and cluster center coordinates (X, Y, Z) of the most significant cluster. Only clusters with >30 voxels were considered. Structures were extracted from the JHU White Matter Tractography Atlas. *L* = left hemisphere, *R* = right hemisphere.

White matter structures that correlated significantly with working memory. Red color indicates voxels for which FA is significantly (TFCE corrected *P* < 0.05) correlated with working memory performance, adjusted for group. *L* = Left, *R* = Right, *P* = Posterior, *A* = Anterior. See Supplementary Figure 1 for the map of the different tracts.

**Fig. 3:**
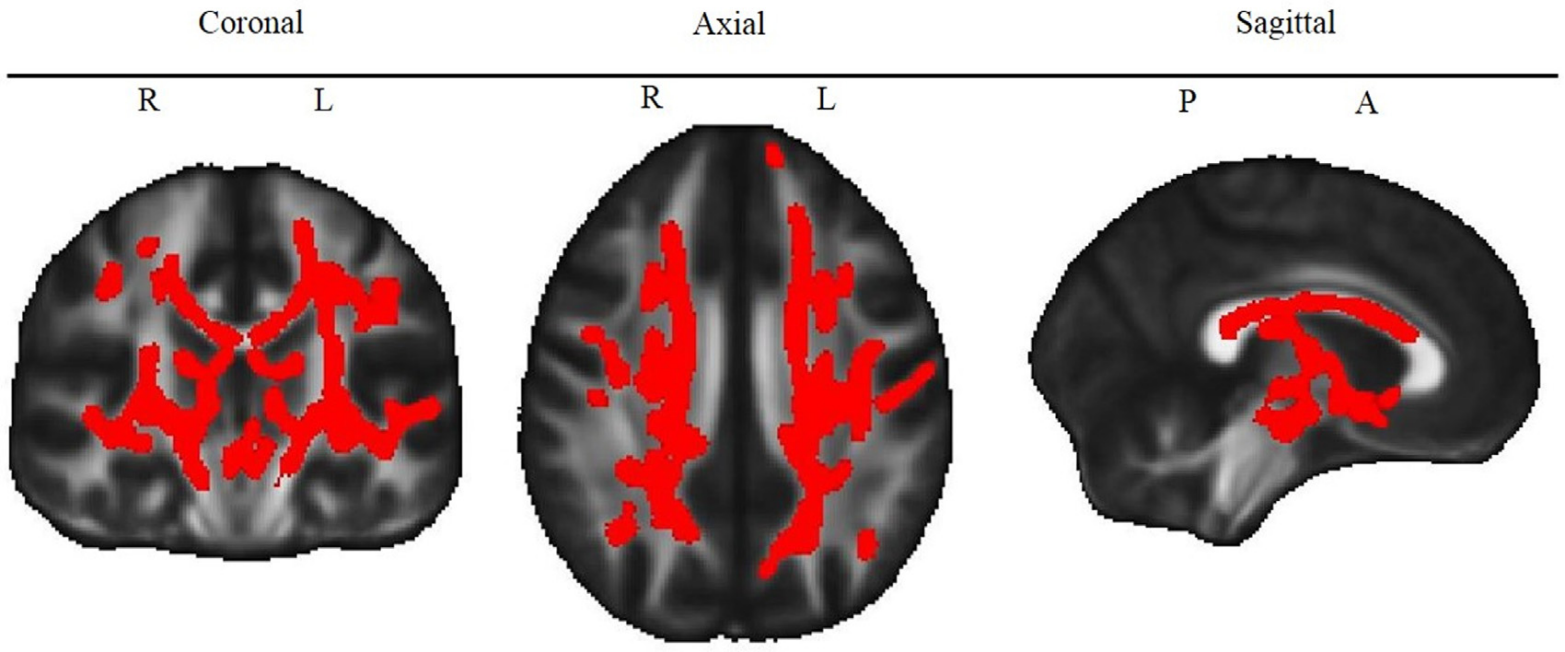
Correlation of FA and working memory in patients only.

A post-hoc TBSS analysis of the link between working memory and FA within the patient and control groups separately revealed a significant association within the patient group but this association did not survive correction for multiple comparisons within the control group. In the patient group, working memory was associated with FA within additional clusters in various bilateral tracts (see Fig. 3), strongly overlapping with the findings from the group comparison (see Fig. 1).

White matter structures that correlated significantly with working memory in the patient subgroup. Red color indicates voxels for which FA is significantly (TFCE corrected *P* < 0.05) correlated with working memory performance in patients only. *L* = Left, *R* = Right, *P* = Posterior, *A* = Anterior. See Supplementary Figure 1 for the map of the different tracts.

A full sample (including both groups) post hoc analysis of the mean FA of all significant clusters (Model 2, see Section 2.4) revealed that the association between working memory and FA was independent of IQ (*β* = −0.000, *t*(80) = −0.479, *P* *=* *=*0.63, *CI-95* = −0.001 to 0.001), age (*β* = 0.003, *t*(80) = 1.292, *P* *=* *=*0.20*, CI-95* = −0.002 to 0.009) and sex (*β* = 0.001, *t*(80) = 0.149, *P* *=* *=*0.88, *CI-95* = −0.016 to 0.018). The association between FA and working memory (*β* = 0.002, *t*(80) = 3.838, *P <* 0.001, *CI-95* = 0.001 to 0.003) remained significant after including these covariates. This model explained 20% of the variance in FA (adjusted *R^2^* = 0.201). The Pearson's correlation coefficient for FA and working memory was *r* = 0.48 (patients only: *r* = 0.46; controls only *r* = 0.47), indicating a moderate effect size.

A third model (Model 3, see Section 2.4) showed no evidence for an interaction between working memory and type of CHD (*β* = 0.001, *t*(43) = 1.060, *P* = 0.30, *CI-95* = −0.001 to 0.003). The strength of the correlation of working memory and FA did not differ between types of CHD (cyanotic, acyanotic).

## 4 Discussion

In this study, we demonstrated altered white matter microstructure in the form of reduced fractional anisotropy (FA) in adolescents with CHD, who underwent cardiac surgery requiring cardiopulmonary bypass during infancy and childhood, compared to healthy controls. Microstructural alterations were widespread and equally detected in patients with cyanotic and acyanotic CHD.  Importantly, white matter FA was associated with impaired working memory performance both in patients and controls in frontal brain areas (bilateral forceps minor and the right frontal part of the uncincate fasciculus and the inferior fronto-occipital fasciculus).

### 4.1 Reduced white matter microstructure in patients with CHD

White matter microstructure was altered in various, bilateral brain regions in CHD patients. Our findings are in line with previous studies reporting microstructural alterations in adolescents with cyanotic CHD, namely patients who underwent the Fontan procedure (Watson et al., 2018) and patients with a corrected transposition of the great arteries (Rivkin et al., 2013). Using similar state of the art DTI acquisition and TBSS methods as Watson and colleagues (Watson et al., 2018), our study underlines that not only patients with a corrected or palliated cyanotic, but also with an acyanotic heart defect show reduced FA across widespread bilateral brain regions. Interestingly, the affected tracts are very similar to those altered in preterm born adolescents (Li et al., 2015; Vollmer et al., 2017). It has been suggested that the cognitive deficits of preterm born children are akin to those in CHD children (Easson et al., 2018). The findings from our current study provide additional evidence for structural similarities between these populations, manifested as changes in FA in the cerebral peduncle, cingulum, posterior corona radiata, corpus callosum, external and internal capsule, forceps minor, fornix, fronto-occipital fasciculus, longitudinal fasciculus, and uncinate fasciculus. However, notable differences are also present, including changes in FA in the anterior and superior corona radiata and the forceps major identified in CHD patients in the current cohort, but not reported in recent preterm studies (Li et al., 2015; Vollmer et al., 2017). Therefore, while these two cohorts may also share underlying neurobiological pathomechanisms, namely alterations in the integrity of white matter microstructure during intrauterine development (Rose et al., 2008), differences in pathophysiology between these populations (Guo et al., 2019) may also lead to alterations in development of the white matter tracts during neonatal period and into adolescence.

There is evidence that microstructural changes are already evident at early life and may persist over time: white matter microstructure have been demonstrated in neonates with CHD (Hagmann et al., 2016; Karmacharya et al., 2018; Mulkey, 2014) and were associated with reduced functional connectivity in EEG (Birca et al., 2016). Zaidi and colleagues (Zaidi et al., 2018) demonstrated that the size of the aorta in neonates with a single ventricle heart defect predicted the anisotropy of the white matter microstructure in adolescents, suggesting a lasting influence of cerebral blood flow on white matter maturation.

### 4.2 Association between white matter microstructure and working memory

The current study provides strong evidence that altered white matter microstructure measured as reduced FA in the bilateral frontal cortex is associated with impaired working memory.

Previous studies in patients with various types of CHD have shown that working memory impairments are present across the lifespan (Bellinger et al., 2015a; Calderon et al., 2010; Ilardi et al., 2017; Murphy et al., 2015; Schaefer et al., 2013). Impaired working memory performance is further associated with attention deficit/hyperactivity disorder (ADHD; Martinussen et al., 2005), which is more prevalent in patients with CHD than in the general population (Hansen et al., 2012; Rollins et al., 2014). However, this has not been assessed in this study. In the school and home settings, working memory difficulties were observed in previous studies by means of the Behavior Rating Inventory of Executive Function (Cassidy et al., 2014; Sanz et al., 2017). The high rate of patients in our study who received school support and completed an additional year of school underlines the academic consequence of cognitive impairments. Thus, it is important to better understand the neural mechanisms underlying working memory impairments in patients with CHD. For instance, reduced whole brain and hippocampal volumes in combination with impaired working memory performance have been described previously in our cohort (Latal et al., 2016; Von Rhein et al., 2014). An fMRI study in a mixed sample of patients with corrected CHD demonstrated alterations of the BOLD signal in the fronto-parietal network of patients with CHD during a working memory task, suggesting a decreased efficiency of this network (King et al., 2016). This interpretation is in line with the current finding of reduced FA in the white matter of the frontal lobe, which may reflect reduced maturation of the inferior fronto-occipital fasciculus, a projection fiber tract connecting the frontal, parietal, and occipital lobe.

The association between working memory and frontal FA was evident in both, left and right hemispheres, which is in line with a recent meta-analysis of fMRI studies demonstrating that both, left and right hemispheric frontal structures are involved in verbal working memory (Emch et al., 2019). Further, the association was independent of group. This is in line with previous findings in typically-developing adolescents showing that increased connectivity between the frontal lobe and parietal/temporal structures is associated with working memory improvement (Krogsrud et al., 2018; Østby et al., 2011; Peters et al., 2012; Vestergaard et al., 2010). Most previous studies investigated only one fronto-parietal tract (i.e. superior longitudinal fasciculus) as a region of interest (Krogsrud et al., 2018; Østby et al., 2011; Peters et al., 2012; Vestergaard et al., 2010), which was not significantly associated with working memory in our cohort. However, the comparison of study results is limited due to different methodology (i.e. age ranges, DTI methods, working memory tasks). Our study revealed that alterations in the inferior fronto-occipital fasciculus, another fronto-parietal tract, the forceps minor (fronto-interhemispheric) and the uncinate fasciculus (fronto-temporal) were associated with working memory. These results were corrected for multiple comparisons and have a medium effect size, indicating robustness of the findings. Future studies should, thus, not only consider the superior longitudinal fasciculus as a region of interest when investigating neuronal correlates of working memory in healthy adolescents but also expand analyses to further tracts connecting the frontal lobe.

Interestingly, the effect size of the association between FA and working memory was comparable between the CHD and control groups (*r* = 0.47, *r* = 0.46 in controls and patients, respectively), but patients showed overall lower FA and lower working memory performance, as well as a wider range of scores. Studies in preterm born adolescents, who are also at risk for working memory impairments, found a similar association between working memory and FA in structures, such as in the forceps minor (Loe et al., 2019; Vollmer et al., 2017). This indicates that neurodevelopmental impairments that are observed in both neonatal at-risk populations, may share underlying structural alterations in the brain. In this regard, future studies are needed comparing neurodevelopmental outcome and brain connectivity between both preterm and CHD populations to better understand its underlying mechanisms.

A post hoc analysis correlating working memory with FA in the patient group separately revealed additional significant clusters in various bilateral tracts, overlapping with the findings of the full group comparison, but no significant association (corrected for multiple comparisons) was observed between FA and working memory within the control group separately. The additional significant structures in patients may reflect a more global effect of neurodevelopmental impairment, such that patients with lower global FA have more neurodevelopmental impairments in various domains, including working memory. However, differences between the findings in patients and controls may also be driven by statistical effects, such as a wider range of working memory scores (and a wider range in FA) in the patients and increased statistical power arising from the larger group size (*N* = 47 patients vs *N* = 39 controls). Since the significant association between working memory and the frontal structures identified in the full sample was independent of group and IQ, after adjusting for those factors, a decrease in frontal FA appears to be specifically linked to impairments in working memory, whereas decreases in FA in other structures, evident in patients only, could potentially reflect a global effect of altered neurodevelopment.

Microstructural maturation is a long-lasting and complex process during childhood and adolescence. Lebel and colleagues (Lebel et al., 2008) have demonstrated that different structures mature at different rates. For instance, most association fibers mature during adolescence, whereas fronto-temporal (e.g. uncinate fasciculus) and subcortical structures (e.g. thalamus) mature later in young adulthood. Moreover, adolescence is a crucial period for the maturation of white matter microstructure in the frontal cortex, which has been associated with improvements in higher order cognitive function, such as working memory (Sousa et al., 2018). Taken together with the known developmental increase in white matter FA throughout adolescence, our findings of FA reduction within frontal tracts in CHD adolescents and its association with working memory impairments, lead to the assumption that the maturation of the frontal cortex might be delayed in adolescents with CHD. Due to the cross-sectional design of this study, implications cannot be drawn as to whether the reduced maturation would remain disrupted after the pubertal maturation process. However, some of the tracts which appear to be altered in adolescents with CHD have been reported to mature late in adolescence (Lebel et al., 2008), and working memory performance gains in importance in this period of life due to increasing demands within the school environment. Adolescence may therefore provide a window of opportunity for cognitive interventions in CHD patients in order to support the process of brain maturation during puberty and thereby increase higher-order cognitive function in these patients.

### 4.3 Limitations

As reported previously, selection bias resulted in an above-average IQ for the CHD patients studied (see Schaefer et al., 2013), which may limit the generalizability of our findings. However, working memory performance was still below average in these patients with overall normal IQ and may be even lower in the general population of CHD patients. Accordingly, impaired white matter maturation may be even more pronounced in the general population of CHD patients.

Interestingly, we did not find any association between working memory performance and FA within the superior longitudinal fasciculus, a prominent structure associated with working memory performance in both, typically developing (Burzynska et al., 2011; Karlsgodt et al., 2008; Østby et al., 2011; Peters et al., 2012; Vestergaard et al., 2010) and in preterm-born adolescents (Vollmer et al., 2017). We may have missed this finding due to our rather conservative correction for multiple testing. However, when investigating CHD patients only, lower FA in the superior longitudinal fasciculus was associated with working memory impairments, indicating that the effect may be stronger in the patients compared to the healthy population.

We were not able to identify any association between white matter microstructure and cardiac risk factors influencing the system perfusion, initial cyanosis or disease severity (i.e. side of CHD, cyanotic CHD, number of surgeries, and age at surgery). Due to the heterogeneity of CHD types included in our study, subgroup comparisons of cardiac risk factors were underpowered. Future studies with well-powered, balanced samples are needed to investigate cardiac risk factors more comprehensively.

In addition, this study used verbal but no visuo-spatial working memory tasks. The most prominent theoretical model of working memory described by Baddeley (2003) suggests that verbal and visuospatial working memory are partially independent processes. Therefore, we cannot infer from the present results that there is any association between visuo-spatial processes and white matter maturation.

## 5 Conclusion

In adolescents with various types of CHD, who underwent cardiopulmonary bypass surgery during infancy and early childhood, widespread bilateral changes in white matter microstructure were observed compared to healthy controls. Furthermore, reduced white matter FA in the frontal lobe was associated with impaired working memory performance in CHD. This association was evident in patients with both cyanotic and acyanotic CHD. The clinical implications of our study are twofold: children with CHD undergoing bypass surgery need to be followed until adolescence and need to be tested for working memory impairments in order to counsel patients, parents and teachers if school problems are present. Secondly, early detection methods and neuropsychological interventions need to be developed to detect and improve these functions in this most vulnerable patient population, helping to improve their academic success and long-term outcome.

## Statements

**Funding:** This work was supported by the Swiss Heart Foundation, Else Kröner-Fresenius Foundation. The sponsors had no influence on study design, the collection, analysis, and interpretation of data, the writing of the report, or the decision to submit the paper for publication.

## CRediT authorship contribution statement

**Melanie Ehrler:** Formal analysis, Writing - original draft. **Beatrice Latal:** Conceptualization, Data curation, Funding acquisition, Methodology, Writing - review & editing. **Oliver Kretschmar:** Methodology, Supervision, Writing - review & editing. **Michael von Rhein:** Conceptualization, Data curation, Funding acquisition, Investigation, Methodology, Writing - review & editing. **Ruth O'Gorman Tuura:** Methodology, Supervision, Writing - review & editing.

## Declaration of Competing Interest

None.

## Appendix Supplementary materials

Image, application 1

Image, application 2
