# Supplementary material for: Altered frontal white matter microstructure is associated with working memory impairments in adolescents with congenital heart disease: A diffusion tensor imaging study
Source: Neuroimage Clin. 2019 Dec 16;25:102123. doi: 10.1016/j.nicl.2019.102123 (PMC6933217; doi:10.1016/j.nicl.2019.102123)
Supplement: Supplementary file 1 [file mmc1.docx]

Supplementary Figure 1: Color coded map of all significant tracts


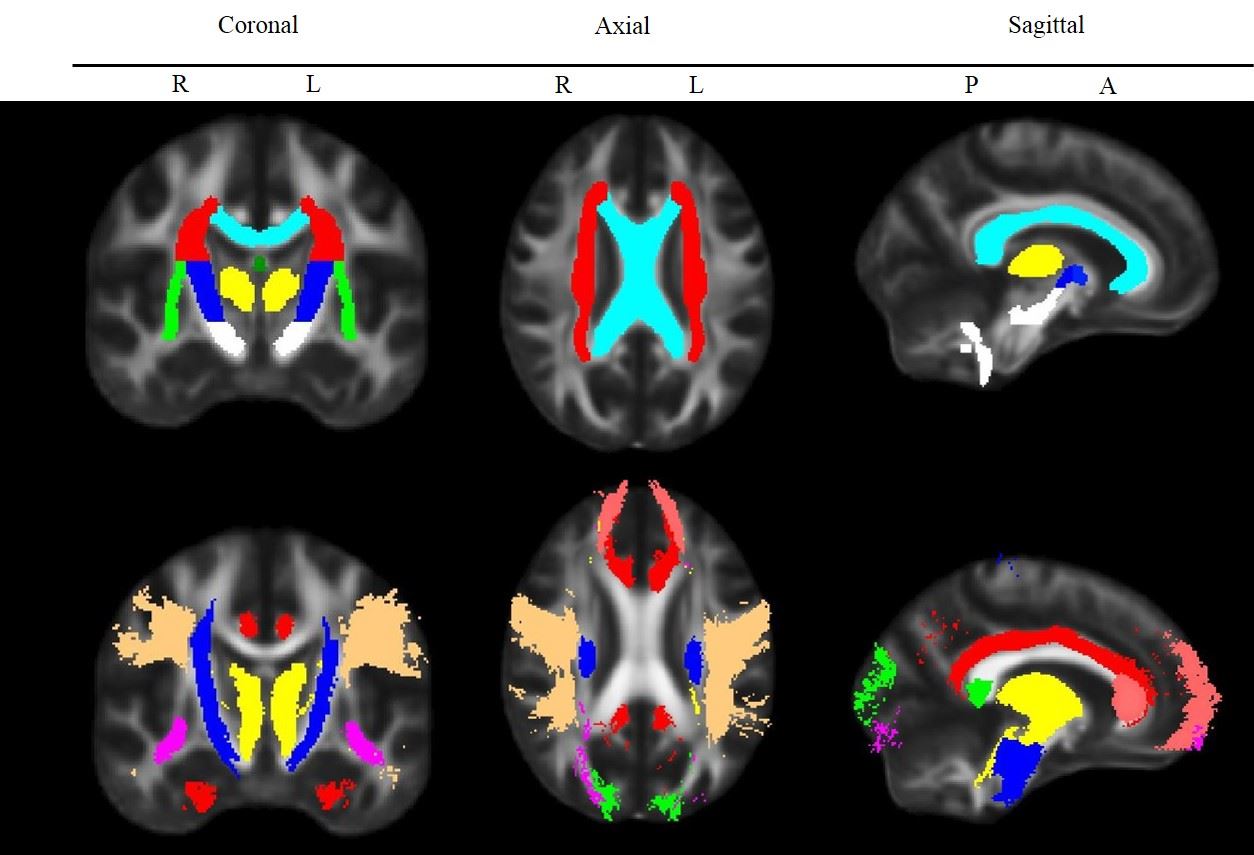


**Upper row:** Tracts extracted from the JHU ICBM-DTI-81 White Matter and the Harvard-Oxford Subcortical Structural atlases. Cerebellar peduncle = white, corona radiate = red, corpus callosum = light blue, external capsule = green, fornix = dark green, internal capsule = dark blue, thalamus = yellow.

**Lower row:** Tracts extracted from the JHU White Matter Tractography atlas. Anterior thalamic radiation = yellow, cingulum = red, corticospinal tract = blue, forceps major = green, forceps minor = salmon, fronto-occipital fasciculus = pink, superior longitudinal fasciculus = coper.
