# Supplementary material for: Altered frontal white matter microstructure is associated with working memory impairments in adolescents with congenital heart disease: A diffusion tensor imaging study
Source: Neuroimage Clin. 2019 Dec 16;25:102123. doi: 10.1016/j.nicl.2019.102123 (PMC6933217; doi:10.1016/j.nicl.2019.102123)
Supplement: Supplementary file 2 [file mmc2.docx]

**Supplementary Table 1: Post hoc analysis on the association between FA and cardiac variables**

| **Cardiac variable** | **Size of subsamples** | ***T*** | ***P*** |
| --- | --- | --- | --- |
| Neonatal CPB surgery (yes, no) | Yes = 9, No = 38 | -0.14 | 0.9 |
| Number of CPB surgeries (1, >1) | 1 = 35; >1 = 12 | 0.58 | 0.6 |
| Side of CHD (left, right, both) | Left = 10; right = 10; both = 26 | -0.1 to -0.8 | all > 0.3 |

Models include mean FA of the significant clusters, obtained from the voxel-wise analysis comparing patients and controls, as dependent variable, cardiac variable as independent variable and sex and age as covariates. Models were calculated for each cardiac variable separately. Transposition of great arteries, Atrial or ventricular septal defect and Truncus arteriosus were classified as both sided CHD.
